# Prevalence of plasmid-mediated AmpC in Enterobacteriaceae isolated from humans and from retail meat in Zagazig, Egypt

**DOI:** 10.1186/s13756-019-0494-6

**Published:** 2019-02-26

**Authors:** Katrijn L. Rensing, H. M. Abdallah, Alex Koek, Gamal A. Elmowalid, Christina M. J. E. Vandenbroucke-Grauls, Nashwan al Naiemi, Karin van Dijk

**Affiliations:** 1 Amsterdam UMC, Vrije Universiteit Amsterdam, Medical Microbiology and Infection Control, Amsterdam Immunity and Infection Institute, Amsterdam, The Netherlands; 2 Department of Microbiology, Faculty of Veterinary Medicine, Zagazig University, Zagazig, Egypt; 3 Laboratory for Medical Microbiology and Public Health, Hengelo, The Netherlands; 4 Microbiology and Infection Control, Ziekenhuisgroep Twente, Almelo, The Netherlands

**Keywords:** Plasmid-mediated AmpC, Group I Enterobacteriaceae, Prevalence, Egypt

## Abstract

**Background:**

The objective of this study was to determine the prevalence of plasmid-mediated AmpC (pAmpC) among Enterobacteriaceae isolated from humans and from retail meat in Egypt.

**Methods:**

Enterobacteriaceae were isolated from patients with suspected bloodstream infection, human fecal samples, retail chicken meat samples and retail sheep meat samples. All group I Enterobacteriaceae were analyzed for presence of pAmpC genes by PCR. Antibiotic susceptibility testing was performed in all pAmpC positive isolates, followed by phenotypic and genotypic ESBL and carbapenemase testing on indication.

**Results:**

The prevalence of pAmpC among group I Enterobacteriaceae isolated from 225 patients with bloodstream infection was 5.6% [95%CI 2.2–13.4]. Among 100 patients with community-onset gastroenteritis the prevalence in fecal samples was 4.8% [95%CI 2.1–10.7]. The prevalence among 112 chicken carcasses and 100 sheep meat samples was 2.4% [95%CI 0.7–8.4] and 1.1% [95%CI 0.2–5.7], respectively. In half of the AmpC positive isolates we detected an ESBL gene and 2 isolates harbored a carbapenemase gene. In five isolates there was resistance to at least three important alternative antibiotic drugs.

**Conclusions:**

We consider the prevalence of pAmpC in Egypt, as found in our study, moderately low. To follow future trends in prevalence of pAmpC worldwide, a standardized screening algorithm for the detection of pAmpC is needed.

## Background

Resistance of Enterobacteriaceae to third generation cephalosporins has become a worldwide problem [1]. The most common cause of resistance to third generation cephalosporins is production of extended-spectrum beta-lactamases (ESBLs). The production of another class of beta-lactamases, the AmpC beta-lactamases (AmpC), also contributes to this problem [2]. This class of beta-lactamases has received less attention initially, but is now increasingly recognized as a growing problem in Enterobacteriaceae.

In several species of Enterobacteriaceae, AmpC is encoded on the chromosome. These species belong to the so-called group II Enterobacteriaceae (like *Enterobacter* spp., *Serratia* spp., *Providentia* spp., *Morganella morganii, Citrobacter freundii* and *Hafnia alvei*) [3]. In these species, chromosomally encoded AmpC can be transcribed at high levels by mechanisms such as induction and derepression, resulting in resistance to third generation cephalosporins [4]. Interestingly, AmpC can also be encoded on plasmids. Plasmid-mediated AmpC (pAmpC) genes are derived from chromosomal AmpC genes of group II Enterobacteriaceae and were initially found in Enterobacteriaceae that lack or have low expression of chromosomally encoded AmpC (like *Klebsiella* spp., *Proteus* spp., *Salmonella* spp., *Escherichia coli*, and *Shigella* spp.), so-called group I Enterobacteriaceae [2, 3].

pAmpC may represent a new threat since plasmid-encoded beta-lactamases are easily transferable between species and can cause nosocomial outbreaks [5–11]. Furthermore, the presence of pAmpC is often associated with multidrug resistance, and moreover, pAmpC in combination with porin loss may lead to resistance to carbapenems [5, 12–15]. Consequently, infections caused by pAmpC-producing Enterobacteriaceae have high therapy failure and mortality rates [5, 16].

The presence of pAmpC-producing Enterobacteriaceae in humans has been reported worldwide, also from several Mediterranean countries, like Spain [17, 18], France [19], Libya [20], Algeria [21], Morocco [22] and Turkey [23]. In addition, pAmpC has been detected in retail meat samples in Mediterranean countries and there are indications that retail animals might form a reservoir for pAmpC-producing Enterobacteriaceae that could be transmitted to humans [24–27].

To date, little is known on the frequency of pAmpC in Egypt. Therefore, the aim of this study was to determine the prevalence of pAmpC among Enterobacteriaceae isolated from humans and from retail meat in Zagazig, Egypt.

## Methods

### Sample collection and bacterial isolation

Bacterial isolates from human bloodstream infections, human fecal samples, retail chicken meat samples and retail sheep meat samples were collected between January 2013 and May 2013, in Zagazig, Egypt. Complete sample collection and bacterial isolation have been described previously [28–30]. Below a summary is provided.

#### Human blood samples

Blood culture samples were obtained from 225 patients with a suspected blood stream infection at El-Ahrar General Hospital, Zagazig, Egypt. In case of growth of Enterobacteriaceae, one isolate per patient was included for further analysis [30].

#### Human fecal samples

Fecal samples were collected from 100 consecutive patients, who presented at El-Ahrar General Hospital, with community-acquired gastro-enteritis. This was defined as community onset of complaints of gastro-enteritis and no prior hospital admission within the previous 10 days before start of the symptoms. Fecal samples (one per patient) were cultured on MacConkey agar [28].

#### Retail sheep meat samples

For a period of 10 weeks, two random meat samples were purchased each week at 5 retail butcher shops in different districts of Zagazig. The samples were immediately transported to the laboratory for culture. Sampling was performed by a swabbing–based method [31]. After collection, each swab was immersed in 5 mL of saline (0.9%), mixed well by vortexing for 10 seconds, and centrifuged at 3,500 × g for 15 minutes. Most of the supernatant was decanted, and 100 μL of the sediment was inoculated directly on MacConkey agar.

#### Retail chicken meat samples

In seven butcher shops, located in different districts of Zagazig City, two random fresh chicken carcasses were bought once a week for a period of 8 weeks. Samples of these carcasses were cultured on MacConkey agar [29].

### Bacterial identification

All morphologically different colonies were identified by the automated Vitek® MS system (BioMérieux, Marcy l’Étoile, France). After identification, Enterobacteriaceae were divided into two groups: group I Enterobacteriaceae, in which inducible or derepressed chromosomal AmpC enzymes are uncommon or absent (like *Escherichia coli, Klebsiella* spp*., Proteus mirabilis, Salmonella* spp., and *Shigella* spp); and group II Enterobacteriaceae, in which inducible or derepressed chromosomal AmpC is common (like *Citrobacter freundii, Enterobacter* spp., *Morganella morganii, Serratia* spp., *Hafnia alvei* and *Providencia* spp.). Two vials of cultured Enterobacteriaceae were lost: one (*R.ornithinolytica*) in the group of patients with gastroenteritis and one (*E.coli*) in the group of chicken meat samples. Therefore, these two isolates are not included in the analysis.

### AmpC PCR

All group I Enterobacteriaceae were analyzed by PCR for molecular detection of pAmpC genes. For this purpose we up-dated the AmpC PCR that was published by Brolund *et al.* in 2010 [32]. This PCR was based on 2 triplet PCR reactions followed by melting point analyses to differentiate between genes belonging to the 6 different AmpC families (CIT, MOX, FOX, ACC, DHA and EBC), as described by Pérez-Pérez [33]. We adapted the primer set in order to also detect more recently described AmpC genotypes (Table 1). The thermal cycling conditions were: 95^0^C for 10 minutes, followed by 35 cycles of 95^0^C for 15 seconds and 65^0^C for 1 minute. Dissociation conditions were 95^0^C for 1 minute, followed by ramping started at 60^0^C for 1 minute continuously increasing towards 95^0^C.

**Table 1:** Overview of primers

Primers	Sequence 5’-3’	Melting point
CIT-F1 CIT-F2 CIT-R1 CIT-R2 CIT-R3	TGG CCA GAA CTG ACA GGC AAA TGG CCT GAA CTG ACT GGT AAG TTT CTC CTG AAC GTG GCT GGC TTT CTC CTG AAC GCG GCT GGC TTT CTC CTG AAC CTG GCT GGC	87.84^0^C
MOX-F MOX-R1 MOX-R2	GCT GCT CAA GGA GCA CAG GAT CAC ATT GAC ATA GGT GTG GTG C CAC ATT GAG GTA GGT ATG GTA C	91.04^0^C
FOX-F FOX-R	AAC ATG GGG TAT CAG GGA GAT G CAA AGC GCG TAA CCG GAT TGG	89.15^0^C
DHA-F DHA-R	AAC TTT CAC AGG TGT GCT GGG T GCT GCC ACT GCT GAT AGA A	88.23^0^C
ACC-F ACC-R	GTG CAA GCC AAT ATG GGG CAG CTC CCA CAT CAG ATC CTG AGT	83.58^0^C
EBC-F1 EBC-F2 EBC-F3 EBC-R1 EBC-R2 EBC-R3	TCG GTA AAG CCG ATG TTG CGG TTG GCA AAG CCG ATA TCG CGG TTG GCA AGG CCG ATA TCG CGG CTT CCA CTG CGG CTG CCA GTT CGC CCA CTG TGG TTG CCA GGA CTT CCA CTG CGG TTG CCA GTG	89.50^0^C

### Antibiotic susceptibility testing and confirmation of ESBL- and carbapenemase production

Antibiotic susceptibility testing was performed for all pAmpC PCR positive Enterobacteriaceae by the automated VITEK®2 system with AST card N198 or N344 (BioMérieux, Marcy l’Étoile, France). The MIC breakpoints according to the EUCAST criteria were used for interpreting the results [34]. ESBL- and carbapenemase production were phenotypically confirmed according to the EUCAST guideline for the detection of resistance mechanisms [35] and the guidelines of the Dutch Society of Medical Microbiology [3], using the ESBL combination disk (Rosco, Taastrup, Denmark) and carbapenemase double disc synergy test [36, 37], respectively.

All phenotypic ESBL- and carbapenemase positive isolates were analysed for the presence of genes encoding *bla*_TEM_, *bla*_SHV_ and *bla*_CTX-M_, and/or *bla*_KPC_, *bla*_NDM_, *bla*_OXA-48_, *bla*_IMP_ and *bla*_VIM_, as previously described [28]. If *bla*_TEM_ or *bla*_SHV_ encoding genes were present in the absence of *bla*_CTX-M_, purified PCR products were sequenced for further analysis [28]. In case of carbapenemase production, and phenotypic ESBL testing could not be interpreted, genotypic ESBL testing was performed, as recommended by EUCAST [35].

### Statistical analysis

Results were expressed as the proportion of group I Enterobacteriaceae that were pAmpC positive. Confidence intervals were calculated using Wilson’s score. Analyses were performed with R package version 3.5.0.

## Results

**Fig. 1:**
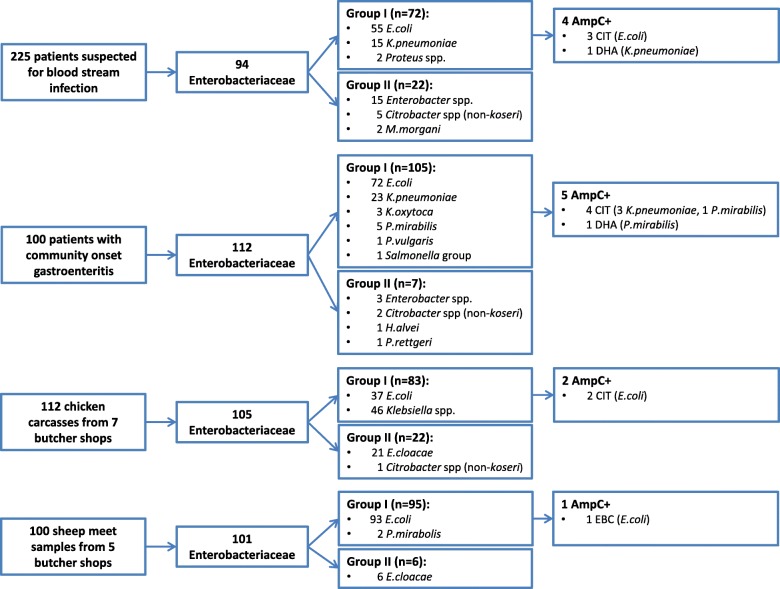
Overview of isolated Enterobacteriaceae

We isolated 94 Enterobacteriaceae from 225 patients with suspected blood stream infection. Seventy-two were group I Enterobacteriaceae of which four were positive in the AmpC PCR [5.6% (95%CI 2.2–13.4)] (Fig. 1 and Table 2). pAmpC was detected in three *E.coli* strains and one *K.pneumoniae* strain. In 100 fecal samples of patients with community-onset gastroenteritis we detected five pAmpC positive isolates out of 105 group I Enterobacteriaceae [4.8% (95%CI 2.1–10.7)]. In this patient group, pAmpC was found in three K.pneumoniae strains and two P.mirabilis strains, all derived from distinctive patients. Overall, three out of 127 clinical *E.coli* isolates (2,4%) and four out of 38 clinical *K.pneumoniae* isolates (10.5%) were pAmpC positive.

**Table 2:** Occurence of plasmidal AmpC in group I Enterobacteriaceae

Population	Proportion of pAmpC+ Enterobacteriaceae	Percentage pAmpC+ (95% CI)
Patients suspected of blood stream infection (*n*=225)	4/ 72 group I Enterobacteriaceae 3/ 55 *E.coli* 1/ 15 *K.pneumoniae*	5,6% of group I Enterobacteriaceae (2,2–13,4) 5,5% of *E.coli* (1,9–14,9) 6,7% of *K.pneumoniae* (1,2– 29,8)
Patients with community-onset gastroenteritis (*n*=100)	5/105 group I Enterobacteriaceae 0/ 72 *E.coli* 3/ 23 *K.pneumoniae* 2/ 5 *P.mirabilis*	4,8% of group I Enterobacteriaceae (2,1–10,7) 0% of *E.coli* (0–5,1) 13% *of K.pneumoniae* (4,5–32,1)
Retail chicken meat (*n*=112)	2/ 83 group I Enterobacteriaceae 2/ 37 *E.coli* 0/ 44 *K.pneumoniae*	2,4% of group I Enterobacteriaceae (0,7–8,4) 5,4% (1,5–17,7) of *E.coli* 0% of *K.pneumoniae* (0–8,0)
Retail sheep meat (*n*=100)	1/ 95 group I Enterobacteriaceae 1/ 93 *E.coli*	1,1% of group I Enterobacteriaceae (0,2–5,7) 1,1% of *E.coli* (0,2–5,8)

Of the 112 chicken carcasses we collected, cultures of 12 carcasses had to be discarded because of growth of *Pseudomonas* spp. (*n*=7) or Gram-positive cocci (*n*=5). In the remaining 100 chicken carcasses, there was growth of 83 group I Enterobacteriaceae, among which two were pAmpC positive [2.4% (95%CI 0.7–8.4)]. In sheep meat, one *E.coli* isolate out of 95 group I Enterobacteriaceae was AmpC positive (1.1% (95%CI 0.2–5.7).

AmpC variants belonging to the CIT family were found most often (*n*=9) followed by variants belonging to DHA family (*n*=2) and EBC family (*n*=1).

Table 3 shows susceptibility patterns and presence of ESBL and/or carbapenemases in all 12 pAmpC encoding group I Enterobacteriaceae. In half of the isolates we detected an ESBL gene. Two isolates were phenotypically ESBL positive, which could not be confirmed genotypically. Two isolates showed carbapenemase production and harbored *bla*_VIM_. Five out of 12 pAmpC encoding Enterobacteriaceae showed resistance to three out of the following antimicrobial categories: aminoglycosides, quinolones, carbapenems and trimethoprim/sulfamethoxazole.

**Table 3:**
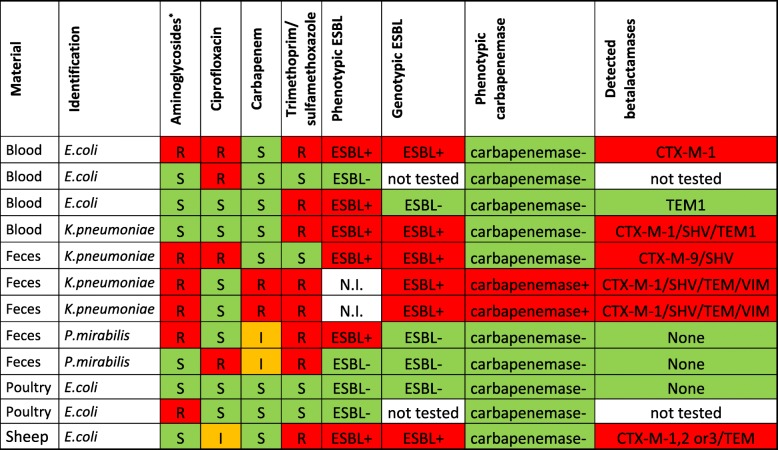
Susceptibility patterns for pAmpC positive group I Enterobacteriaceae

*R* resistant, *S* susceptible, *I* intermediate susceptible, *N.I.* not interpretable, *+* positive, *–* negative

Red colour: resistance (mechanism) detected. Green colour: no resistance (mechanism) detected, orange colour: intermediate susceptibility detected.

^*^gentamicin and/or tobramycin

## Discussion

The prevalence of pAmpC in group I Enterobacteriaceae isolated from humans and from retail meat in Egypt varied between 1% and 6%. Nearly 5% of Egyptian patients with community-onset gastroenteritis were colonized with pAmpC-producing group I Enterobacteriaceae. In patients with suspected blood stream infection, the prevalence of pAmpC among group I Enterobacteriaceae isolated from blood was comparable. In contrast, Helmy *et al.* found a prevalence of pAmpC of 16% among group I Enterobacteriaceae that were isolated from Egyptian hospitalized patients with a urinary tract infection [38]. This higher prevalence might be explained by a difference in patient populations: Helmy *et al*. collected samples from hospitalized patients, including ICU patients, whereas in our study samples were derived from both hospitalized and non-hospitalized patients. As suggested by Helmy *et al.*, the hospitalized patients in their study may have recently been exposed to cephalosporin therapy, which has been described as an independent risk factor for infection with pAmpC-producing Enterobacteriaceae [39]. Another study that has been conducted in Egypt, among healthcare workers of an Egyptian hospital, showed a prevalence of 3%: out of 200 health care workers, six were colonized with pAmpC producing *E.coli* [40]. As a reference, in other North African countries, prevalence of pAmpC in humans varied between 2.3% of Enterobacteriaceae in Algeria [21] and 6.7% of *E.coli* in Libya [20].

In Europe, the prevalence of pAmpC seems a bit lower than the prevalence we found in Egypt. In the Netherlands, the prevalence among non-hospitalized subjects has been reported to be 0.6% [41] and 1.3% [42]. The same prevalence of 0.6% has been reported among Spanish patients [17]. When focusing at species level, studies in Denmark [43], France [19] and Czech Republic [44] showed that respectively 0.06%, 0.09% and 1.3% of clinical *E.coli* isolates were pAmpC positive. For *K.pneumoniae* this was 0.5% of *K.pneumoniae* in Czech Republic [44] and 3% of *K.pneumoniae* in Ireland [45]. In our study, in Egypt, these percentages were higher: 2,4% of clinical *E.coli* isolates and 10.5% of clinical *K.pneumoniae* isolates were pAmpC positive. In Asia, the percentage of clinical *E.coli* isolates that is pAmpC positive varies over a wider range. The lowest prevalence has been reported in studies in Japan (0.12% and 1.7% of *E.coli* [46, 47]), Iran (2.8% of *E.coli* [48]) and China (2% of *E.coli* [49, 50]). In a study in Turkey, 10.9% of clinical *E.coli* isolates carried pAmpC genes [23]. The highest prevalence was found in India: of the 132 *E.coli* isolates, that were acquired from unique hospitalized patients in six distant hospitals in India, 60 were positive in the pAmpC PCR, resulting in a prevalence of 45.5% [51]. In this study, the prevalence among *K.pneumoniae* was also high (29.4%), whereas in other studies in Asia the prevalence of pAmpC among *K.pneumoniae* was 10% or less [23, 46, 49, 50].

When evaluating the currently available literature discussed above, the prevalence of pAmpC of around 5%, as found in our study, is similar to the prevalence as found in other North African countries (ranging from 2.3%-6.7%). It should be mentioned, however, that in general, data regarding prevalence of pAmpC are difficult to compare, since studies use different methods for the detection of pAmpC. First of all, screening methods for detection of suspected pAmpC-producing isolates differ widely. Secondly, PCR-based confirmation methods vary. Many studies used the PCR as initially described by Pérez-Pérez in 2002 [33]. At that time, 29 AmpC genes within 6 AmpC families were known. In the past few years, however, the number of known AmpC genes has increased. Therefore, in our study, we used a PCR that aimed to detect 183 AmpC genes. Consequently, previous studies might have missed AmpC genes that were not yet discovered at that time or that were not targeted by the used AmpC PCR. Additionally, some of the studies did not aim to detect AmpC genes of all six AmpC families. In some studies only PCR was done for detecting members of the CIT family, or the focus was only on detecting *bla*_*CMY*_*.* Furthermore, in some studies the sample sizes were small, resulting in large 95% confidence intervals. Therefore, there are several limitations when comparing studies on prevalence of pAmpC. Still, we consider the prevalence of pAmpC among Egyptian patients, as found in our study, moderately low. As a reference, we previously showed that prevalence of ESBL among Enterobacteriaceae recovered from patients with community-onset gastroenteritis and blood stream infection in Egypt was much higher: 65% and 49% respectively [28, 30].

We also determined the prevalence of pAmpC among Enterobacteriaceae recovered from Egyptian retail meat. We found that between 1% and 2.5% of group I Enterobacteriaceae isolated from retail sheep and chicken were pAmpC positive. Compared to other studies, this is low. Previous studies have shown a prevalence of pAmpC in retail chicken of 9-12% [24–27]. Whether it is likely that retail meat in Egypt forms a reservoir for pAmpC producing Enterobacteriaceae cannot be deducted from our study and needs further investigation. If, however, pAmpC would spread from meat for consumption to humans it will be important to take adequate measures in livestock breeding, such as minimizing consumption of antibiotics in animals and proper food preparation.

Most of the pAmpC positive isolates in our study (9 out of 12) contained AmpC genes belonging to the CIT family. This is in concordance with studies all over the world, showing that CMY-2 is the most prevalent pAmpC gene [15, 19, 24, 26, 38, 52, 53]. We found two DHA positive isolates, which have most often been detected in Asia [46, 49]. The less common EBC variant was detected only once in our study. Compared to the study by Helmy *et al.*, which was also conducted in Egypt, genotypic distribution among pAmpC positive isolates was about the same, although they also detected three genes that belonged to the MOX family and three genes that belonged to the FOX family [38].

It is known that plasmids that encode AmpC genes often carry many other resistance genes [12, 15, 54, 55]. Indeed, we found that almost half of the plasmid-encoding AmpC Enterobacteriaceae showed resistance to three out of the four most important alternative drug choices for treating infections with pAmpC producing Enterobacteriaceae: aminoglycosides, ciprofloxacin, carbapenems and trimethoprim/sulfamethoxazole. Resistance to trimethoprim/sulfamethoxazole was seen most often, in 8 of 12 pAmpC positive isolates. Carbapenem resistance was seen in four isolates, in which in two cases it was caused by carbapenemase production. In the other two cases it might have been caused by the concomitance of pAmpC with porin loss [5, 13, 14]. This co-existence of pAmpC and resistance to other alternative antibiotic therapy is worrying; it is likely to cause therapy failure since few or no therapeutic options are left.

Although the prevalence of pAmpC in Egypt is still relatively low, the occurrence of pAmpC in humans and in meat for consumption poses a possible threat comparable to ESBL. Currently, international guidelines do not provide recommendations for the detection of pAmpC in routine laboratory practice. Therefore, in most laboratories, pAmpC producers will not be recognized. This might have consequences for the further spread of pAmpC. Indeed, it has been shown that prevalence of pAmpC has increased over time [49, 56]. This increase, together with co-existence of resistance to important alternative antibiotic therapies, forms a potential threat. We believe that, for better understanding the occurrence and consequences of pAmpC, standardized screening algorithms for the detection of pAmpC are required.

## Conclusions

Currently, standardized screening algorithms for the detection of pAmpC are lacking. Therefore studies that describe the prevalence of pAmpC use different detection methods and consequently are difficult to compare. Based on the current literature, we consider the prevalence of pAmpC in Egypt, as found in our study, moderately low. In order to be able to follow future trends in the prevalence of pAmpC worldwide, a standardized screening algorithm for the detection of pAmpC is necessary.

## Abbreviations

AmpC: AmpC betalactamases
ESBL: Extended-spectrum beta-lactamase
pAmpC: Plasmid-mediated AmpC
